# Improving public health sector service delivery in the Free State, South Africa: development of a provincial intervention model

**DOI:** 10.1186/s12913-022-07777-x

**Published:** 2022-04-12

**Authors:** Benjamin Malakoane, James Christoffel Heunis, Perpetual Chikobvu, Nanteza Gladys Kigozi, Willem Hendrik Kruger

**Affiliations:** 1 Department of Community Health, University of the Free State, PO Box 339, Bloemfontein, 9300 South Africa; 2 Centre for Health Systems Research & Development, University of the Free State, PO Box 339, Bloemfontein, 9300 South Africa; 3 Department of Community Health, Free State Department of Health, University of the Free State, PO Box 277, Bloemfontein, 9300 South Africa

**Keywords:** Free State, WHO health systems building blocks, Health system strengthening, Integration, Governance and Accountability

## Abstract

**Background:**

Public health sector service delivery challenges leading to poor population health outcomes have been observed in the Free State province of South Africa for the past decade. A multi-method situation appraisal of the different functional domains revealed serious health system deficiencies and operational defects, notably fragmentation of healthcare programmes and frontline services, as well as challenges related to governance, accountability and human resources for health. It was therefore necessary to develop a system-wide intervention to comprehensively address defects in the operation of the public health system and its major components.

**Methods:**

This study describes the development of the ‘Health Systems Governance & Accountability’ (HSGA) intervention model by the Free State Department of Health (FSDoH) in collaboration with the community and other stakeholders following a participatory action approach. Documented information collected during routine management processes were reviewed for this paper. Starting in March 2013, the development of the HSGA intervention model and the concomitant application of Kaplan and Norton’s (1992) Balanced Scorecard performance measurement tool was informed by the World Health Organization’s (2007) conceptual framework for health system strengthening and reform comprised of six health system ‘building blocks.’ The multiple and overlapping processes and actions to develop the intervention are described according to the four steps in Kaplan et al.’s (2013) systems approach to health systems strengthening: (i) problem identification, (ii) description, (iii) alteration and (iv) implementation.

**Results:**

The finalisation of the HSGA intervention model before end-2013 was a prelude to the development of the FSDoH’s Strategic Transformation Plan 2015–2030. The HSGA intervention model was used as a tool to implement and integrate the Plan’s programmes moving forward with a consistent focus on the six building blocks for health systems strengthening and the all-important linkages between them.

**Conclusion:**

The model was developed to address fragmentation and improve public health service delivery by the provincial health department. In January 2016, the intervention model became an official departmental policy, meaning that it was approved for implementation, compliance, monitoring and reporting, and became the guiding framework for health systems strengthening and transform in the Free State.

## Background

The Free State province of South Africa has recorded substandard population health outcomes at least since 2011 [1]. The dire need for effective and implementable interventions to improve public health services and outcomes is clear [2]. To avoid resource wastage, efforts should be made to systematically design health systems strengthening interventions with the potential of system-wide implementation [3–5].

When a new Member of the Executive Council (first author) assumed leadership of the Free State Department of Health (FSDoH) in 2013 there was a clear need to address the rising burden of both communicable and non-communicable disease [6], as well as declining community trust in the public health system. The Member of the Executive Council outlined a vision to “strengthen the health system’s effectiveness, drive system changes, improve burden of disease outcomes, [and] ensure financial sustainability for better health outcomes and increased life expectancy.”

A multi-method situation appraisal of the FSDoH’s public health services was undertaken by the first author, a senior programme manager (third author) in collaboration with two health systems researchers (second and fourth authors) and a community health specialist (fifth author) in collaboration with healthcare workers in 2013. The appraisal established that the main overall system challenges underlying sub-standard performance in the province included fragmentation of services, staff shortages, financial/cash-flow problems and leadership and governance defects [1]. There was a clear need to improve programme integration and leadership, and to address organisational, human resources and financial deficiencies in this setting. The above-mentioned team collaborated in developing, implementing and evaluating a whole-system intervention to improve public health service integration and outcomes, namely the Health Systems Governance & Accountability (HSGA) intervention.

Although no universal definition or concept of integration exists [7], in 2005, the World Health Organization (WHO) explained the term as follows: “The notion of integration has a long history. Integration is supposed to tackle the need for complementarity of different interdependent services and administrative structures, so as to better achieve common goals. In the 1950s these goals were defined in terms of outcome, in the 1960s of process and in the 1990s of economic impact” [8 pp. 108–109]. A systematic review conducted in 2016 defined integration as “a variety of managerial and operational changes to health systems that bring together inputs, delivery, management, and organizations of particular service functions, in order to provide clients with a continuum of preventative and curative services, according to their needs over time and across different levels of the health system” [9 p. 2]. The WHO suggests that integration has to take place at three levels: (i) patient level, i.e., case management; (ii) service delivery level, i.e., multiple interventions provided through one delivery channel and (iii) systems level, i.e., bringing together the management and support functions of different sub-programmes, and ensuring complementarity between the different levels of care [8]. Integration is a choice that affects programme financing, planning and delivery, and, ultimately, the achievement of public health goals [10, 11].

Integration of disease control programmes such as the HIV/AIDS, tuberculosis and chronic disease programmes within the district health system and comprehensive primary health care (PHC) approach has been a priority in South Africa for decades [12, 13]. However, attempts at integration are hindered by leadership and management challenges as observed in three provinces in the complex dynamics between managers responsible for specific policies and those responsible for managing the integrated delivery of all policies [14].

The WHO [2, 15] advances that health systems are comprised of six ‘building blocks’ that need to be considered in health system strengthening: (i) service delivery; (ii) health workforce; (iii) information; (iv) medical products, vaccines and technologies; (v) financing and (vi) leadership and governance (stewardship). The building blocks framework has been found to be useful to assess in-country health system performance [16–18]. However, there is some uncertainty as to how the framework can best be used to address the problems of fragmentation and ineffective performance of public health systems in low- and middle-income countries. Mounier-Jack et al. [19] thus recommended that researchers using the building blocks framework should adapt it and make it context-specific.

The FSDoH leadership believed that conceptualisation of the public health system in terms of the building blocks framework and improved integration of service programming and rendering through, amongst others, reconfigured facility clusters and service hubs, would help to address a broad spectrum of health system challenges. The decision was therefore made to design and develop a system-wide re-engineering intervention to address the fragmentation of healthcare provisioning using a systems approach. The working definition for a systems approach was one that “applies scientific insights to understand the elements that influence health outcomes; models the relationships between those elements; and alters design, processes, or policies based on the resultant knowledge in order to produce better health at lower cost” [20 p. 4].

Health systems are complex and characterised by high levels of variability, uncertainty, and dynamism [21]. Kaplan et al. (2013) [20] proposed that the use of a systems approach is beneficial in addressing complex health systems challenges by considering the dynamic interaction among the multiple elements (i.e., people, processes, policies, and organisations) involved in caring for patients and the multiple factors influencing health. These authors proposed four essential considerations or steps in designing interventions from the perspective of a systems approach, (i) identification, (ii) description, (iii) alteration and (iv) implementation. These steps were followed in the development of the HSGA intervention model in the Free State. Kaplan and Norton’s (1992) Balanced Scorecard performance measurement tool [4] was concomitantly put in use.

Details on the implementation and assessment of the intervention including the results of a questionnaire survey and focus group discussions with health managers and community representatives will be reported elsewhere. This descriptive study relates the development of an intelligible and promising intervention, the HSGA intervention model, and its formalisation into an official policy, the HSGA policy, to address fragmentation and improve public health service delivery in the Free State by the provincial health department in collaboration with its stakeholders. A participatory design involving a wide range of public health stakeholders and role players in plans and efforts to improve the integration and outcomes of public health services was followed as part of routine health service delivery management processes.

## Methods

### Setting

The FSDoH serves a population of approximately 2.9 million people [22], of whom about 80% are public health sector dependent [23]. The province includes four district municipalities, Fezile Dabi, Lejweleputswa, Thabo Mofutsanyana and Xhariep, and one metropolitan municipality, Mangaung. In 2015/16, PHC was provided by 211 fixed PHC clinics, ten community health centres and numerous mobile clinics [6]. Hospital services included 24 district hospitals, four regional hospitals, one specialised psychiatric hospital, one tertiary hospital and one central hospital. The PHC clinics, community health centres and hospitals respectively provide primary, secondary, and tertiary care services. Hospitals are managed by chief executive officers with hospital boards providing oversight or governance. PHC clinics and community health centres are managed by operational managers reporting to their respective district offices. Clinic committees provide oversight or governance and report to the provincial executive authority. District health managers are responsible for planning and monitoring of disease control programme implementation within their districts, with District Clinical Specialist Teams providing supportive supervision, clinical governance, and attending to health systems and logistics, staff development and user-related considerations [24].

### Design

The development of the HSGA intervention model followed a participatory action approach [25–27] as part of routine health service delivery management processes which took place from 2013 to 2015. The reports and minutes generated during these routine management processes were reviewed using the WHO health systems building blocks framework to conceptualise the intervention. This approach was chosen as it has been shown that interventions developed using participatory designs are more likely to be acceptable and implemented effectively [28, 29]. A province-wide consultative process was embarked upon, wherein communities and stakeholders within the public health system were called upon to comment on the different functional domains: (i) the skill base of the management; (ii) patient and clinical workflow; (iii) the referral system and (iv) leadership alignment to community and operational needs, process reliability, and attainment of the desired outcomes [1]. The involvement of both managers and communities in shaping the HSGA intervention model empowered the executive authority to lead health system change and administrative action [30]. The designations of 584 FSDoH functionaries, community representatives, and other stakeholders who partook in the development of the model as part of routine health service delivery management processes from 2013 to 2015 are indicated in Table 1.

**Table 1:** Designations of the HSGA intervention model development participants

Designation	Number
Executive leaders (Member of the Executive Council, Head of Department, 2 Deputy Director-Generals and the Chief Financial Officer)	5
District managers	5
Hospital chief executive officers	24
Programme directors	42
District Clinical Specialist Team directors	2
Deputy directors	8
Clinic managers	189
District Management Team members	182
District Clinical Specialist Team members	18
PHC clinic committee & hospital board members	84
Partners/non-governmental organisations	14
Provincial cabinet member	1
Health Professions Council of South Africa representative	1
Labour organisation representatives	2
Academics	3
Traditional leader	1
Traditional healers	3
Total	584

### Key activities in the development process of the HSGA intervention model

**Fig. 1:**
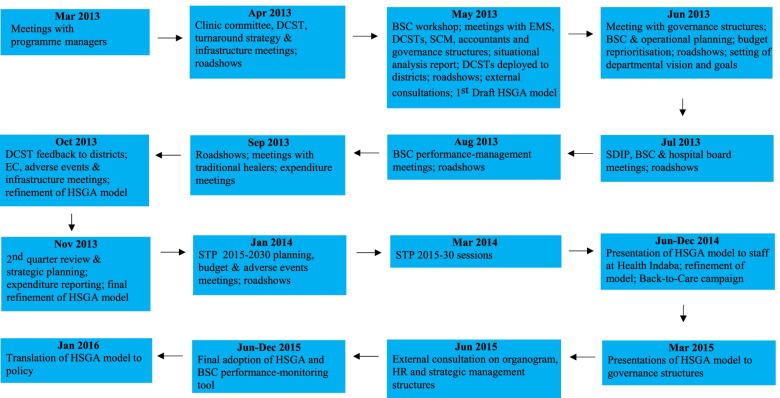
Key activities in HSGA intervention model development *BSC* Balanced Scorecard, *DCST* District Clinical Specialist Team, *EMS* emergency medical services, *EC* Executive Committee, *HR* human resources, *MEC* Member of the Executive Committee, *PHC* primary health care, *SCM* supply change management, *SDIP* Service Delivery Improvement Plan, *STP* Strategic Transformation Plan

The chronology of key activities in the development of the HSGA intervention model is depicted in Fig. 1.

During March–April 2013, the executive management held meetings with PHC clinic committees and infrastructure improvement teams to understand the healthcare challenges faced by catchment communities, as well as the state of PHC infrastructure. Given that key health systems challenges had been identified in the preceding multifaceted situational analysis [1], the community engagements were aimed at teasing out specific malleable challenges from a wide range of health systems problems facing the province. During the same period, and for the same reason, hospital and PHC clinic ‘roadshows’ (organised campaigns carried out in identified catchment areas led by the executive leadership) commenced and turnaround strategy discussions were held at the corporate level.

During May 2013, in a quest to assess the status and quality of Emergency Medical Services, the referral system and patient and clinical workflow, as well as the management skill base and the budget support received in doing their work, various meetings were held with Emergency Medical Services, District Clinical Specialist Teams, supply chain management and governance structures in order to understand the status quo and leadership alignment to community and operational needs, process reliability, identification of areas needing intervention, and attainment of the desired outcomes.

Hospital and PHC clinic roadshows continued, and the first Balanced Scorecard workshop was conducted. Often applied to healthcare settings and organisations [31–33], the Balanced Scorecard performance measures include four questions or perspectives: (i) how do customers see us? (customer perspective); (ii) what must we excel at? (internal perspective); (iii) can we continue to improve and create value? (innovation and learning perspective) and (iv) how do we look to shareholders? (financial perspective) [4]. The Balanced Scorecard was adopted to enable production of evidence and tracking of progress when reporting. The Balanced Scorecard workshop culminated in the development of the first draft of the HSGA intervention model accompanied by relevant policy and procedure reviews to introduce a culture of coordination and communication within the confines of policy provisions.

The executive authority then presented the findings of the previous three months to the corporate and district-level managers with new mandates being given to various staff components. The District Clinical Specialist Teams were specifically responsible to visit PHC clinics and hospitals in their respective districts to conduct surveillance and monitoring of effectiveness of clinical protocols. They were to complete the investigation of the challenges and to provide recommendations within five months.

The PHC clinic committee and hospital board (governance) structures are by their nature, community advocacy formations. Meetings with them continued into June-July 2013 in tandem with the hospital and PHC clinic roadshows. A plan to operationalise the Balanced Scorecard was drafted, and budget reprioritisation sessions held. Additional meetings took place to discuss ways to conform to the new Service Delivery Improvement Plan and the Balanced Scorecard approach. Besides the continuation of hospital and PHC clinic roadshows, Balanced Scorecard and expenditure reporting meetings were held during August–September 2013 to discuss ways to improve programme performance.

During October–November 2013, the District Clinical Specialist Teams had returned from their five-month expedition into the districts. They provided feedback covering the sub-districts visited and problem areas identified. The HSGA intervention model was then refined to address the shortcomings identified and relevant policies and procedures were amended accordingly. After the second quarter performance review process and expenditure reporting, the model was further refined to streamline the interventions with policy and procedure reviews.

During January-March 2014, the Strategic Transformation Plan 2015–2030 [34] processes related to format, programmes and content were commenced, and relevant functionaries invited with clear mandates to present their ideas on what could further be done to improve service and programme delivery to the executive and senior management, including programme managers. Thereafter, the hospital and PHC clinic roadshows were completed in the last of the districts. The draft HSGA intervention model was also presented to the staff and stakeholders at a province-wide health ‘*indaba*’ (collective discussion or meeting). Thereafter, the model was further refined together with concomitant changes to policies and procedures.

During March-December 2015, the HSGA intervention model was presented to governance structures and across the whole department to progressively introduce the new way of working, which would inform the performance assessment system according to the Balanced Scorecard approach as well. External consultants were involved in helping to refine the strategic management issues and in assisting the streamlining of the organogram to address the changes to be implemented. The Balanced Scorecard was thus used during performance assessments to test compliance with the needed changes.

Finally, in January 2016, the HSGA intervention model became an official departmental policy [35]. The intention of the HSGA policy was to ensure the sustainability of implementation of the HSGA intervention model to strengthen compliance and entrench a culture of using the model as an operational system to achieve the aims of the Strategic Transformation Plan 2015–2030. Information gathered in the above processes was synthesised using an iterative participatory design approach with three main steps: (i) consultations, (ii) incorporating suggestions and (iii) revisions. Repeated consultations with a broad range of stakeholders (Table 1) took place where inputs were sought, and changes incorporated as part of health services delivery management process. By doing so, the HSGA intervention model was refined at each consultation point. Final revision followed an *indaba* with the executive leadership, District Management Teams, community representatives, departmental line managers, governance structures, labour organisations, academics, traditional leaders and healers, and PHC clinic committees. They proposed changes to clusters and service hubs and focused on ensuring that the content of the intervention model was appropriate and consistent with the National Department of Health’s (NDoH) guidelines as this would allow for easier embedding of the model into routine care and was likely to make it more acceptable to healthcare managers and workers.

### Development of the intervention

The development of the HSGA intervention model is subsequently related according to the four steps outlined by Kaplan et al. [20].

#### Step 1: Identification

Kaplan et al. [20 p. 5] define this step as follows: “Identify the multiple elements involved in caring for patients and promoting the health of individuals and populations.” In the context of the development of the HSGA intervention model, this implied identifying all those elements involved in service delivery whose improvement would lead to improved integration and health outcomes. The identification of the multiple health system factors contributing to poor health service delivery was described by Malakoane et al. [1] and further investigated during the model development activities (Fig. 1).

In summary, a multi-method situation appraisal based on analysis of 44 reports generated in 2013 through presentations by unit managers, sub-district assessments by District Clinical Specialist Teams, and group discussions with district managers, PHC clinic supervisors and managers, and chief executive and clinical officers at all hospital levels was conducted. The appraisal established that the rising burden of disease and poor health outcomes were principally due to extant fragmentation of health service delivery and the disjointed way in which the public health system was operating. Other problems included poor policy and regulatory coordination, verticalisation of programme organisation and administration, staff shortages, budgetary problems, and, notably, leadership and governance deficits, as significant health system challenges that resulted in ineffective public healthcare service delivery [1].

Specific challenges with respect to people, processes and policy shortfalls within the health system are depicted in Table 2 in line with the WHO health systems building blocks framework. All the elements involved in service delivery that were investigated during the model development activities (Fig. 1) and whose improvement would lead to improved integration and health outcomes were consolidated in reports and minutes. These reports and minutes of the meetings were then thematically synthesized.

**Table 2:** Building blocks, people, process, and policy shortfalls

Building block	People	Process	Policy shortfalls
Service delivery	Communities & staff unhappy about quality of services	• Long waiting times • Medicines inadequate • Facilities untidy • Non-adherence to infection control measures • Staff attitude negative	• Core standards not implemented & monitored
Health workforce	Staff dissatisfied with working conditions	• Critical vacancies • Qualified staff not appropriately placed • Staff work in ‘silos’ • Poor performance & evaluations	• Policies & delegations unknown & flouted • Staffing norms not implemented
Information	Inadequate data capturers & unfilled posts	• Information services for monitoring trends unreliable & not used for planning & decision-making • Insufficient access to hardware & connectivity in facilities	• Information management & information technology policies not adequate
Medicines, products & equipment	Staff non-compliant with stock taking & drug usage tracking	• Procurement processes inefficient • Drug stocks ran out with no buffer stock • Essential medical technology non-functional • Stock orders not aligned to usage • No stock tracking system in place	• Weak supply chain management policy & lack of monitoring by facility cost centres
Finance	Finance personnel lacked fiduciary responsibility	• Financial resources not allocated to impactful programmes • Prioritised programmes not budgeted for • Unfunded mandates prioritised • Irregular & unauthorised expenditure incurred	• Weak financial management policies, delegations & practices
Leadership/governance	Lack of initiative & duty of care among senior managers	• No budget priorities • Weak programme monitoring & reporting • No coordination of policy & procedure • Lack of accountability • Financial & human resource delegations flouted	• Poor consequence management • Weak referral policy & coordination

#### Step 2: Description

**Fig. 2:**
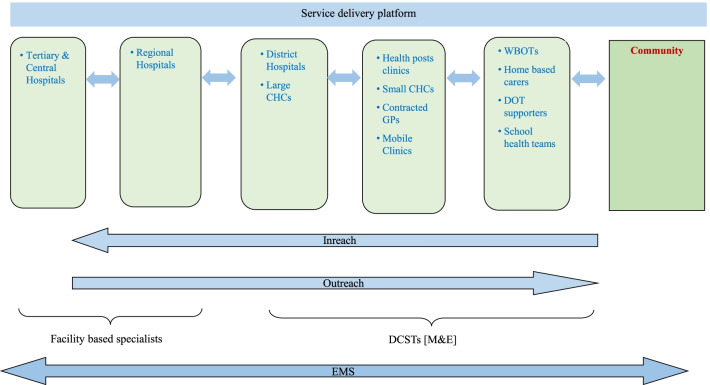
Service delivery platform ‘Inreach’ refers to referral to a higher level of care. *CHC* community health centre, *DCST* District Clinical Specialist Team, *DOT* directly observed treatment, *EMS* emergency medical services, *GP* general practitioner, *M&E* monitoring and evaluation, *WBOT* Ward Based Outreach Team

Kaplan et al. [20 p. 5] defines this step as follows: “Describe how those elements [aspects involved in caring for patients and promoting the health of individuals and populations] operate independently and interdependently.” The decision to reconfigure the public healthcare delivery platform for better integration of services and improved population-health outcomes was inevitable and required an intelligible and feasible intervention. In view of the multiple challenges that were identified (Table 2), and the extent to which continuation of these challenges impacted or infringed patients’ rights, the strengthening of the public health system was approached by means of systematic application of the WHO’s building blocks framework. It was particularly important to bring about a well-planned and managed referral system supported by policy and regulation and to deploy adequate resources across all levels of care [36, 37]. The HSGA intervention model was intended to address, interlink and integrate all the public health service delivery processes for better outcomes (Fig. 2).

The executive leadership convened all the corporate managers, district managers, programme managers, hospital chief executive officers and clinic managers (Table 1) to discuss the findings of the situation appraisal. These discussions focused on required alterations to improve the public health system’s performance and advance cooperation between facilities and functionaries to enable integration of healthcare service delivery in consideration of the six WHO health system building blocks. It was agreed that a system to integrate the service delivery platform had to be developed. The executive leadership revisited and outlined the vision and trajectory for this, with a view to identify possible changes including goals aimed at achieving better health outcomes.

The vision for the required reconfiguration of the health system provided by the leadership was to: “increase life expectancy through health system effectiveness, driving system change and ensuring sustainable quality services.” Implementation of the intervention was expected to achieve seven departmental goals whose achievement would lead to the expected health outcomes envisaged during the intervention formulation: (i) provision of strategic leadership and creation of a social compact for better health outcomes; (ii) managing the financial affairs for sustainable health service delivery; (iii) building a strategic and dedicated workforce that is responsive to service demands; (iv) re-engineering PHC to improve access to quality services; (v) developing, operating and managing infrastructure for compliance and better health outcomes; (vi) strengthening the information and knowledge management system to optimise performance and research capabilities and (vii) optimising and supporting implementation of key priority programmes (transformation, affirmative action and business process re-engineering).

The main factors to be addressed included mainstreaming of the WHO health systems building blocks, strengthening of policies and procedures, re-engineering of patient admission and discharge, and improving the leadership capacity of managers. Amongst the malleable factors included were aspects forming part of the managers’ additional duties and reporting responsibilities, firstly, in terms of the service delivery building block, improved integration; and, secondly, in respect of the leadership/governance building block, appropriate distribution of delegated powers within the system.

Emergency Medical Services are crucial in ensuring consistent accessibility of the different levels of healthcare through the referral system. There was a need to review the referral policy and guidelines and to reconfigure referral pathways to enable easier and quicker access to services with the referral of patients to the closest health facility in times of need and to reduce the number of patient transport and emergency vehicles on the road so as to reduce operational costs.

Reconfiguration of the health service platform was to entail, firstly, that the fixed PHC facilities had to be grouped into clusters such that each cluster would consist of a community health centre or 24-h clinic with a number of fixed PHC clinics, mobile clinics, health posts, and Ward Based Outreach Teams referring to it within a given vicinity and not necessarily aligned to municipal boundaries. The composition of these teams is six to ten community health workers, a data capturer and a team leader. In terms of South Africa’s PHC re-engineering agenda [38], these teams are meant to become part of the multi-disciplinary PHC team setup within the district health system. The health promotion, disease prevention, therapeutic, rehabilitative and palliative roles of the Ward Based Outreach Teams must be supported by health practitioners in PHC facilities and by environmental health officers in the community [39]. The teams are managed by professional or enrolled nurses who are additionally responsible for post-natal and community health worker-referred home visits to patients.

The second aspect of the reconfiguration of the health service platform was that each PHC cluster was to fall under the leadership and supervision of one cluster manager and management team and each PHC clinic or community health centre had to be managed by one operational manager. Smaller district hospitals within a specific district were to be reorganised as complexes managed by a chief executive officer with a standardised management team, including clinical, nursing service, administration and finance managers. The chief executive officers of the hospital complexes were to report to the chief directors responsible for the different districts.

The third aspect of the reconfiguration of the health service platform was that the District Clinical Specialist Teams had to assume a leadership role in clinical governance programmes in district hospitals in conjunction with the hospital clinical managers.

#### Step 3: Alteration

Kaplan et al. [20 p. 5] defines this step as follows: “Change the design of organizations, processes, or policies to enhance the results of the interplay and engage in a continuous improvement process that promotes learning at all levels.” The executive leadership decided that a skills audit needed to be conducted and to revisit and revise the FSDoH’s organogram. A decision was made to redeploy some managers to more suitable areas of operation and to revise their responsibilities. These changes were to be supported by compliance to the public service regulations of which the executive authority was the custodian. The intention was to ensure that the right people were placed in the right positions to enable them to improve their performance and to help the FSDoH deliver better public health service outcomes. The whole process of altering the previous ways of working was informed by the conceptualisation of the health systems building blocks and interlinking change mechanisms championed by executive functionaries as shown in Table 3. It was also decided to revisit and amend the previous Strategic Transformation Plan of 2012–2030 on an annual basis to keep functionaries engaged in continuous service improvement on a sustainable basis using the HSGA intervention model.

**Table 3:** Change mechanisms per health system building block

Building block	Change mechanism	How change was affected
Service delivery	• Conduct process flow mapping & integration • Enforce compliance with norms & standards • Reconfigure service platform • Re-engineer patient admission (READ) • Re-engineer patient discharge (RED) • Complex district hospitals & cluster community health centres & primary health care clinics • Establish health posts in community • Reduce waiting times • Improve availability of medicines • Improve facility cleanliness • Improve infection control • Improve patient & staff safety • Improve staff attitudes • Improve Emergency Medical Services response times	• Managers developed flow maps • Compliance included as key performance area • Challenges in every section identified • Admission patterns & policies revised • Discharge patterns & policies revised • Patient-facility ratios considered • Health posts were included in infrastructure budget • Appointment system was introduced & staff increased • Common medicine procurement prioritised • Staff hiring & procurement of equipment & material expedited • Personal protective equipment procured & disinfection increased • Ward security & access control improved • Incentive scheme developed & incentives awarded • Tracking system installed on all ambulances
Health workforce	• Fill vacant & critical vacancies • Appoint staff on merit & skill • Improve teamwork • Conduct workshops on policies & HR delegations • Enforce compliance & performance monitoring	• Critical posts identified & advertised • Staff job profiles & performance assessed • Constant team building exercises • Workshops scheduled & monitored • Key performance areas revised & monitored
Information	• Maintain integrity of health information for monitoring trends, planning & decision-making • Improve access to internet connectivity in facilities & maintain 99% ‘uptime’	• Internet connectivity installed & hardware bought • Reliable service provider was contracted
Medical products, vaccines & technologies	• Improve supply chain management • Prevent drug stockouts & maintain buffer stock • Maintain essential medical technology • Align stock ordering with facility headcounts • Monitor implementation of stocktaking system	• Changed lead & turnaround times • Implemented daily stocktaking • Proactive maintenance programme developed • Alignment of stocks & numbers done • Weekly system-based stock level reporting introduced
Financing	• Allocate financial resources for impactful programme implementation • Stop implementation of unfunded mandates • Implement prudent expenditure management practices	• Aligned the budget to prioritised strategic programmes • Implemented in-year monitoring & reporting • Implemented monthly expenditure reporting
Leadership/governance	• Develop vision of organisation • Introduce priority setting linked to budget & organogram • Inculcate evidence-based decision-making • Foster monitoring & evaluation culture • Strengthen policy & procedure coordination • Consequence management for poor or failed implementation • Allocate & monitor implementation of financial & HR delegations • Implement inreach & outreach capacity-building programmes	• Vision analysed & changed • Strategy developed & linked to budget • Culture of management by risk implemented • Monthly feedback/reporting meetings introduced • Compliance to policy included as a key performance area • Deviations/exceptions reported & addressed • Role clarifications performed & delegations reviewed • Arrangements for inreach & outreach programmes made with relevant level managers

The finalisation and refinement of the HSGA intervention model by end-2014 was a prelude to the development of the Strategic Transformation Plan 2015–2030 in January 2014. The model was to be used as a tool to integrate and implement the Plan’s programmes moving forward. Hence, the HSGA model was approved as an official policy for compliance, monitoring, and reporting in January 2016. The HSGA policy would guide the FSDoH’s approach in implementing the reconfigured service platform and improving healthcare service integration, governance and accountability. It was expected that the HSGA policy would ensure uniform understanding and application of the HSGA intervention model and performance measures of the Balanced Scorecard throughout the department’s services.

#### Step 4: Implementation

Kaplan et al. [20 p. 5] defines this step as follows: “Operationalize the integration of the new dynamics to facilitate the ways people, processes, facilities, equipment, and organizations all work together to achieve better care at lower cost.” The HSGA policy outlined the different implementation roles and responsibilities of the various managers, institutions, stakeholders and formations within the FSDoH and aligned all the annual performance plans, district health plans and the Strategic Transformation Plan 2015–2030 to the HSGA intervention model using the Balanced Scorecard as a performance management tool.

The incremental implementation of the HSGA intervention model which took place from February 2014, was to ensure sustainable implementation and compliance to policy and procedure and was based on normalisation process theory. The implementation process was also directed by the six WHO health systems building blocks that were translated into the seven departmental goals. A subsequent paper will assess the impact of the HSGA policy on the public health systems functioning from the viewpoint of health managers and governors based on Balanced Scorecard performance measurement results.

## Results

The HSGA intervention model was developed and formalised into policy in a quest to bind the employees and managers across the whole department to comply with implementation of the model in a sustainable manner and in line with the Strategic Transformation Plan 2015–2030. The model was designed with support and input from all the relevant stakeholders.

**Fig. 3:**
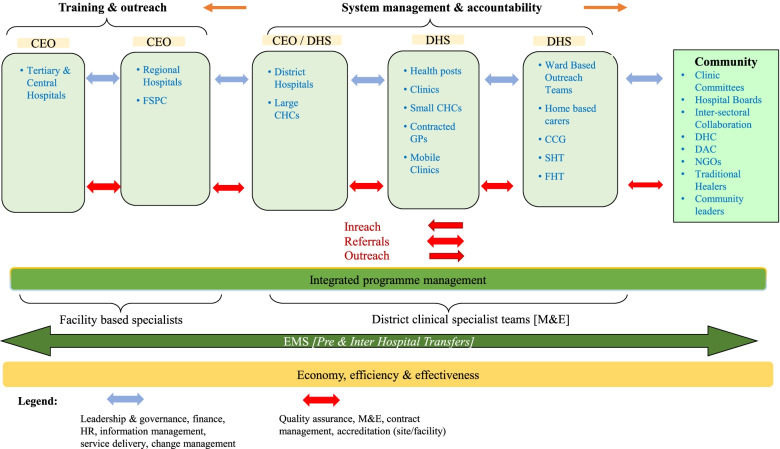
Health Systems Governance & Accountability intervention model ‘Inreach’ refers to deploying health professionals to a higher level facility for purposes of learning. ‘Outreach’ refers to deploying health professionals to a higher level facility for purposes of teaching. *CCG* community care giver, *CEO* chief executive officer, *CHC* community health centre, *DAC* District AIDS Council, *DHC* District Health Council, *DHS* district health system, *EMS* Emergency Medical Services, *FHT* Family Health Team, *FIT* Facility Improvement Team, *FSPC* Free State Psychiatric Complex, *GP* general practitioner, *M&E* monitoring and evaluation, *NGO* non-governmental organisation, *SHT* School Health Team

A process flow was conceptualised on how service delivery at the district level could be functionally integrated with the next levels of referral through a cascade termed Re-engineering of Admission (READ) to enable smooth referral of the patient from the community up to the tertiary level of care. Another process flow termed the Re-engineering of Discharge (RED) [40] was adopted to direct the safe referral and movement of the patient downwards through the system. These changes were reflected in a conceptual framework, the HSGA intervention model, illustrating the need for interventions to integrate the various levels of care within the reviewed policy positions and structural rearrangements (Fig. 3).

The HSGA intervention model emphasised the accountability of the system to the community and patients it served. It indicated how the different healthcare levels dovetailed into one integrated system to improve health system performance based on the six health system building blocks. It provided a framework for leadership, governance, and accountability throughout the whole health system, including community- and facility-based PHC, as well as district, provincial, tertiary, and central hospital services. All healthcare providers were required to comply with the approved referral and service protocols and to provide feedback to the referring facility clinician for further handling and down-referral for continuity of care.

In respect of the service delivery building block, the HSGA intervention model and policy also directed that all public healthcare facilities had to adhere to the norms and standards required by the Council for Health Service Accreditation of Southern Africa; healthcare services had to be provided in an integrated manner with good co-operation and communication between facilities and functionaries; patient movement up and down the health service value-chain had to be managed in line with procedural and clinical protocols; monitoring and evaluation functions had to be co-ordinated by the strategic planning and monitoring and evaluation unit at the corporate office; the District Clinical Specialist Teams had to conduct clinical oversight, clinical governance protocol audits and surveillance in district facilities; and the different levels of care had to be linked through a reciprocal transfer and referral system.

Regarding the leadership/governance building block, the HSGA intervention model and policy directed that leadership and managers had to ensure coordination and integration of governance efforts around a common mission to focus management teams’ activities on achieving optimum performance of the public health system; adopt a holistic view of the system’s operations to enable effective achievement of desired outcomes; comply with service integration imperatives and clinical governance protocols; and monitor obstacles with immediate intervention where necessary. Led by the district managers, the District Management Teams were responsible for governance at the PHC level, integration of service implementation, and compliance with policies and procedures. The District Management Teams were expected to collaborate with multiple internal and external stakeholders who play a role in disease control programme management. All the changes were streamlined in accordance with the building blocks and outlined in the draft Strategic Transformation Plan 2015–2030 [41].

Table 4 shows the changes in the configuration of the institutions per district, including the planned establishment of 32 health posts. A health post is a facility below the level of a clinic which is staffed by lower-category nurses allowing community members to collect their chronic medicines or receive care for minor ailments like wound care. Comprehensive PHC services provided by the Ward Based Outreach Teams in the households, health posts, mobile clinics and fixed PHC facilities were established for effective and efficient PHC re-engineering, thus bringing healthcare services closer to the people. The PHC facilities were re-organised into PHC clusters, each having a group of PHC clinics and a community health centre. Certain PHC clinics were upgraded to community health centres, and certain small hospitals downgraded to community health centres.

**Table 4:** Reconfigured service platform

Health District	Facility type	Previous no	Planned no
Fezile Dabi	Health posts	0	9
	Mobile PHC clinics	12	21
	Fixed PHC clinics	33	30
	Community health centres	5	10
	District hospitals	4	4
	Regional hospital	1	1
Lejweleputswa	Mobile clinics	14	11
	Health posts	0	3
	Fixed PHC clinics	44	39
	Community health centres	1	8
	District hospitals	5	3
	Regional hospital	1	1
Mangaung Metro	Mobile clinics	10	10
	Health posts	0	5
	Fixed PHC clinics	42	41
	Community health centres	2	4
	District hospitals	3	3
	Tertiary hospital	1	1
	Central hospital	1	1
	Specialised hospital	1	1
Thabo Mofutsanyana	Mobile clinics	23	24
	Health posts	0	11
	Fixed PHC clinics	73	66
	Community health centres	1	10
	District hospitals	9	3
	Regional hospitals	2	2
Xhariep	Mobile clinics	12	13
	Health posts	0	4
	Fixed PHC clinics	20	19
	Community health centres	1	3
	District hospitals	3	2
Free State total	Mobile clinics	71	79 (+ 8)
	Health posts	0	32 (+ 32)
	Fixed PHC clinics	212	195 (-17)
	Community health centres	10	35 (+ 25)
	District hospitals	24	15 (-9)
	Tertiary hospital	1	1
	Central hospital	1	1
	Specialised hospital	1	1

EMS are responsible for all transportation of patients, both in emergencies and planned patient referrals in FSDoH facilities. Patient routes had to be aligned with the changed referral paths and routes according to the reviewed departmental referral policy considering that the 60-seater buses were to be operated on long distances to Bloemfontein only. Bloemfontein is the capital of the province and is where most of the higher-level referral and specialist hospitals are located. The categories of patients referred to Bloemfontein was reviewed in line with the approved service packages and patient needs. The new routes improved the flow of patients to the nearest facilities with reduced transit times. Therefore, the operational costs of Emergency Medical Services were reduced as well.

To transform mental health service delivery by 2030, the service delivery system focused on the following aspects within an agreed upon timeframe. In line with the WHO (2003) recommendations [42] regarding organisation of mental health services, the mental health system was planned to include an array of settings and levels that included primary care, community-based settings, general hospitals, and specialised psychiatric hospitals. It was further planned that the Free State Psychiatric Complex would undertake outreach visits to regional hospitals and that the latter would in turn conduct outreach services to district hospitals and selected community health centres. Further outreach services would be provided to the Kimberley Hospital Complex in the Northern Cape province and Queen Mamohato Memorial Hospital in Lesotho.

## Discussion

This study describes the development the Health Systems Governance & Accountability (HSGA) intervention model by the FSDoH in collaboration with the community and other stakeholders following a participatory action approach. Researchers publish the processes they use to develop interventions to improve healthcare to help future developers to improve their approaches, plans and practices. Description of the development of interventions is important because it provides future developers with the terminology and language to identify, conceptualise and explain their rationales and more effectively plan and design context-specific intervention models [43]. It is important to understand how the models that end up being designed during interventions, complement the traditional ways of doing things for the betterment of a common good such as public health. A movement toward an assets-based approach to intervention design and implementation has started to take place in public health where intervention development processes emphasise activating and drawing on the strengths and skills of individuals, communities, and through the co-production of intervention programmes and research [44].

A systematic review of studies reporting intervention development showed that they mainly took a pragmatic self-selected approach, a theory- and evidence-based approach, or a partnership approach, e.g., community-based participatory research or co-design [45]. Both theory (systems) and provider/user approaches were utilised in the development of the HSGA model in the Free State. Systems theory and the WHO health systems building blocks framework helped the developers and participating stakeholders to conceptualise and plan a whole-system intervention, the HSGA intervention model and policy. The participatory approach allowed the developers to incorporate the experiences and ideas of frontline health systems users and providers and managers across all levels of the public health hierarchy. Obtaining both user and provider perspectives is crucial in efforts to model and design context-specific public health strengthening initiatives [46–48].

Previous studies to find ways to improve health outcomes through a consultative and iterative process involving users identified barriers such as weak and unorganised referral patterns and inadequate human resources as crucial [44, 49]. The development of the ‘Better Health Outcomes through Mentoring and Assessment’ (BHOMA) intervention in Zambia was intended to specifically address such challenges [17]. Similar challenges were addressed in the development of the Free State HSGA model, but the focus was on improving integration and performance assessment as means to improve health outcomes. Both interventions were intended to provide the system-wide solutions to the challenges, rather than focusing on specific diseases. The influence that the users exerted could be thought of as a ‘user intervention’ because they would in turn support changes and improvements while constantly assessing whether they are appropriate or not. Von Hippel [44] and Altman et al. [50], reiterate that the context and support for this participatory approach to ‘user intervention’ leverages user innovation capability in influencing the manner of medicine practice or provision of health services, which is considered a novel approach in dispensing public health. Von Hippel explains that when faced with problems or challenges, the users of the products, services or beneficiaries of processes are often likely to develop solutions to their problems and sometimes offer innovative ways and new approaches and solutions to challenges facing them. Hence the essential aspects of the HSGA intervention model were premised on collective solution finding and co-creation of approaches. Users and communities therefore got to enjoy the benefits of the changes they themselves helped to bring about. That is why integration of inputs from users or beneficiaries into the development of innovative approaches or models should be considered as a way to leverage the strengths of communities served by health providers for efficient and effective interventions to be developed and new approaches to be generated to address the varied complexities of health service challenges they face [50].

In the development of the HSGA intervention model obtaining users and communities’ perspectives took the form of unannounced visits to hospitals and clinics and community meetings respectively. This worked well because during the unannounced visits and community meetings, different and diverse groups of patients and providers tended to congregate. They presented their respective views and suggestions on which processes need to be changed or improved for the service offering to improve. This was challenging to deal with because the views and recommendations of the respective users, communities and frontline health staff were often diverse and needed to be deciphered, understood by all parties, and reconciled.

In the development of the HSGA intervention model obtaining managers and providers’ perspectives took the form of learning from and incorporating their views and suggestions presented during routine management meetings, as well as engagements during routine performance assessments. This was advantageous because challenges and proposed solutions presented by the managers and providers could then be directly discussed and addressed. Defects in the referral processes and clinical procedures were identified and reviewed for translation into corrective policy positions. This was challenging because procedures and processes often had to be reviewed ‘on-the-go’ and drafters were to affect the changes on the existing process and procedure documents forthwith, for later implementation.

In the development of the HSGA intervention model obtaining other stakeholders and experts’ perspectives took the form, amongst others, of a health *indaba*. This was beneficial because various stakeholders at the *indaba* raised their views, some of which corroborated the issues earlier raised by users and communities, as well as during the initial situation appraisal. It was sometimes difficult to differentiate personal experiences from health system shortcomings relating to process or procedure defects or challenges. Nevertheless, the recommendations made were considered in the overall revisions of processes and procedures.

Using systems theory to understand organisational performance helps intervention developers to obtain a broader perspective that enables recognition of patterns, cycles and overall structures, rather than focus on specific events or aspects of the system [51, 52]. While functional integration of health services may be an attainable vision [53], certain important intersecting health system capabilities are required to bring about frontline service integration as well. These include fully functional frontline health services, adequately trained and motivated healthcare workers, availability of suitable technology, and devolving authority and decision-making processes to lower cadres of frontline managers and staff to adapt integration processes to local circumstances [54]. Therefore, during the development of the HSGA model, Kaplan’s theory influenced the assessment of how the health system was organised, as well as the identification of the various service interlinkages and interdependencies of the multiple elements (building blocks) involved in public health systems strengthening and performance. The various interlinkages and process dependencies were clearly described, broadly understood and reconfigurations entrenched through policy changes to ensure continuous improvement of the Free State public health system’s performance.

The WHO considers systems thinking as an essential ingredient in opening pathways to identification and resolution of health system challenges [55, 56]. The use of the WHO building blocks approach [2, 15] to guide the integration process of the HSGA intervention model, placed emphasis on the interactions and interdependencies to guide the management of transitions among and between the demand side (community) and the supply side (healthcare facilities and staff).

The Zambian BHOMA study [17] proposed a framework to evaluate a complex health system intervention applying systems thinking concepts. It formed the basis for intervention development using systems thinking, where emphasis was placed on understanding the complete system and how the various parts interacted with each other to create a functional whole as opposed to the individual parts that made up the system [57]. In the development of the HSGA model in the Free State, system theory and the WHO building blocks framework were used to as a ‘template’ to assess the effectiveness and operational efficiencies of various components or divisions within the health services platform. This approach was appropriate because we could discern areas of weakness and how they could be addressed.

The HSGA intervention model was directed at three levels of care, i.e. (i) the community and sub-district levels of care; (ii) the district and regional level of care and (iii) the provincial level of care. At the community and sub-district level, care was to be delivered by Ward Based Outreach Teams and community health workers; at the district or regional level, by local PHC clinics, community health centres and district and regional hospitals; and at the provincial level, by provincial hospitals and the central hospital. Within and between these levels, transitions in the form of READ and RED [40] were intended to be managed efficiently and effectively in an integrated manner to mitigate morbidity and mortality. Austin et al. [49] acknowledged that the movement of a patient from the home or community setting into the health system during admissions into a healthcare facility or hospital and vice versa, constitutes transitions to avoid the effects of care-risk factors that can lead to morbidities, excess stays and even re-admissions if those transitions are ineffective and inefficient. In the Free State, the READ and RED transitions were conceptualised and applied to improve referral up and down the referral hierarchy. The effectiveness and efficiency of these transitions were based on the strength of the referral system which the HSGA intervention model sought to integrate and improve. Austin et al. [49] pointed out that both the system and patient factors exert influence on the transitions and the capacity to address complex barriers to safe admission and discharge procedures and workflows. The development of approaches like the HSGA intervention model to address and improve complex transitions effectively and efficiently require continued testing and assessment of their effectiveness in view of the complexity of the transitions.

Austin et al. [49] corroborate the importance of iterative, internally driven intervention, monitoring, and wide dissemination during the implementation process as was applied in the Free State and will be reported in another paper. In the current study monitoring or evaluation was conducted using Kaplan and Norton’s (1992) Balanced Scorecard performance measurement tool [4]. This decision was based on its successful application in healthcare settings and organisations elsewhere [31–33]. Our experience aligns with that of Oliveira et al. [31] that the Balanced Scorecard is a valuable management tool to enhance flexibility, collaboration, innovation and adaptation that contribute to improve healthcare integration and outcomes.

A limitation of this descriptive study is that it is first authored by the former executive leader of the FSDoH, and an element of bias is thus inevitable. However, inclusion of authors who are not employees of the department helped to mitigate this bias. A strength of the current study is that the health system as a whole, and not just disparate elements within it, were considered in the development of the HSGA intervention model. The study provides useful insight on practical issues to consider during the development of public health interventions geared towards health systems strengthening and moves away from the traditional practice of addressing only individual behaviour change. Furthermore, this study attempts to provide a systematic approach to health systems intervention development with community and stakeholder involvement and aligns with Wight et al.’s [3] and Kaplan et al.’s [20] recommendation that a systematic approach to intervention development, as well as rigorous evaluation, are required. Another strength of the HSGA intervention model is that it was developed during normal service delivery processes and mostly during routine management planning meetings and discussions.

## Conclusion

Public health practice should be evidence-based and grounded in theory and systems thinking. The WHO’s health system building blocks framework is a useful guide to conceptualise and assess the status of a health system and its performance and is a valuable tool to align or configure services according to communities’ actual realities and needs. Based on the building blocks concept, the HSGA intervention model was developed by the provincial health department during routine service delivery conditions to bring about desired reforms where gaps and inefficiencies had previously been identified. Reconfiguration of the service platform provided an enabling framework for future efforts to enhance integration, improve service delivery and improve the public health system’s performance. Thus, the development of the HSGA intervention model and its formalisation into an official policy provided an opportunity to improve healthcare service integration, health system performance, and improve health outcomes in the Free State public health sector. The study contributes to the body of knowledge on health systems strengthening and illustrates how to draw on both theory and participatory approaches to develop an intelligible intervention to improve public health system integration and performance.

## Data Availability

No datasets were generated during the documentary review and therefore no data sharing is applicable to this article.

## Abbreviations

BHOMA: Better Health Outcomes through Mentoring and Assessment
FSDoH: Free State Department of Health
HSGA: Health Systems Governance & Accountability
NDoH: National Department of Health
PHC: Primary health care
READ: Re-engineering of Admission
RED: Re-engineering of Discharge
WHO: World Health Organization
